# 756 Self-Inflicted Frostbite with Dry Ice: A Case Report

**DOI:** 10.1093/jbcr/irac012.309

**Published:** 2022-03-23

**Authors:** Sarah K Shingleton, Michael G Chambers, Michael R Rowland, Garrett W Britton, Anthony P Basel

**Affiliations:** USAISR, San Antonio, Texas; USA ISR Burn Center, Fort Sam Houston, Texas; Brooke Army Medical Center, Trauma Department, Universal City, Texas; US Army Institute of Surgical Research, JBSA Fort Sam Houston, Texas; United States Army Institute of Surgical Research, Fort Sam Houston, Texas

## Abstract

**Introduction:**

Frostbite (FB) is a severe form of cold injury that may result in significant morbidity. Freezing causes formation of ice crystals and protein denaturation leading to cellular damage and tissue necrosis. Although commonly affecting populations who live and work or become stranded in cold environments, the true prevalence is unknown due to lack of standardized reporting. Dry ice is a solid form of carbon dioxide commonly found in the food industry, that can reach temperatures as cold as -110 degrees Fahrenheit and can be purchased at local department stores. Exposure to dry ice is an unusual cause of FB injury that could occur anywhere, especially in warmer climates. Healthcare professionals practicing in warmer climates may be unprepared to assess and treat patients with such injuries.

**Methods:**

We describe a case of self-inflicted FB injury to the bilateral lower extremities (BLE) following intentional submersion in dry ice who presented to a Southern U.S. Burn Unit.

**Results:**

A 20-year-old man with self-diagnosed Body Integrity Identity Disorder (BIID) sustained a 12% TBSA FB injury to his BLE following intentional submersion in dry ice for an estimated 4 hours (Figure 1). He presented to the Burn Center from an outside facility approximately 12 hours post injury with initial rewarming having already occurred. He immediately underwent catheter directed intra-arterial thrombolysis with tissue plasminogen activator (TPA) for leg length preservation. TPA infusion was initiated at 16 hours post-injury for a total infusion time of 24 hours. Wound care consisted of gentle twice daily cleansing with silver sulfadiazine and mafenide acetate creams. The patient ultimately required bilateral below the knee amputations which successfully healed with minimal autografting to the distal limbs. He was formally diagnosed with schizophrenia by our behavioral health team and initiated on risperidone. He underwent aggressive physical therapy and was discharged home with his parents after 21 days. He underwent weekly mental health follow up appointments and has been seen regularly in our burn and rehabilitation clinic.

**Conclusions:**

This unusual case is one of few to describe Grade 4 FB after exposure to dry ice and highlights that severe FB may occur in any climate and can cause severe morbidity. Severe cold injury management is important for burn care professionals to maximize tissue and limb preservation.

